# Cardiac and Skeletal Muscle Transcriptome Response to Heat Stress in Kenyan Chicken Ecotypes Adapted to Low and High Altitudes Reveal Differences in Thermal Tolerance and Stress Response

**DOI:** 10.3389/fgene.2019.00993

**Published:** 2019-10-11

**Authors:** Krishnamoorthy Srikanth, Himansu Kumar, Woncheoul Park, Mijeong Byun, Dajeong Lim, Steve Kemp, Marinus F. W. te Pas, Jun-Mo Kim, Jong-Eun Park

**Affiliations:** ^1^Animal Genomics and Bioinformatics Division, National Institute of Animal Science, RDA, Wanju, South Korea; ^2^Animal Biosciences, International Livestock Research Institute (ILRI), Nairobi, Kenya; ^3^Wageningen UR Livestock Research, Animal Breeding and Genomics, Wageningen, Netherlands; ^4^Department of Animal Science and Technology, Chung-Ang University, Anseong, South Korea

**Keywords:** heat stress, hub genes, PPAR signaling, MAPK signaling, p53 signaling, RNA-Seq

## Abstract

Heat stress (HS) negatively affects chicken performance. Agricultural expansion will happen in regions that experience high ambient temperatures, where fast-growing commercial chickens are vulnerable. Indigenous chickens of such regions, due to generations of exposure to environmental challenges, might have higher thermal tolerance. In this study, two indigenous chicken ecotypes, from the hot and humid Mombasa (lowland) and the colder Naivasha (highland) regions, were used to investigate the effects of acute (5 h, 35°C) and chronic (3 days of 35°C for 8 h/day) HS on the cardiac and skeletal muscle, through RNA sequencing. The rectal temperature gain and the number of differentially expressed genes (DEGs) [False Discovery Rate (FDR) < 0.05] were two times higher in the acute stage than in the chronic stage in both ecotypes, suggesting that cyclic exposure to HS can lead to adaptation. A tissue- and stage-specific difference in response to HS was observed, with peroxisome proliferator-activated-receptor (PPAR) signaling and mitogen-activate protein kinase (MAPK) signaling pathways, enriched in heart and skeletal muscle, respectively, and the p53 pathway enriched only in the acute stage in both tissues. The acute and chronic stage DEGs were integrated by a region-specific gene coexpression network (GCN), and genes with the highest number of connections (hub genes) were identified. The hub genes in the lowland network were *CCNB2*, *Crb2*, *CHST9*, *SESN1*, and *NR4A3*, while *COMMD4*, *TTC32*, *H1F0*, *ACYP1*, and *RPS28* were the hub genes in the highland network. Pathway analysis of genes in the GCN showed that p53 and PPAR signaling pathways were enriched in both low and highland networks, while MAPK signaling and protein processing in endoplasmic reticulum were enriched only in the gene network of highland chickens. This shows that to dissipate the accumulated heat, to reduce heat induced apoptosis, and to promote DNA damage repair, the ecotypes activated or suppressed different genes, indicating the differences in thermal tolerance and HS response mechanisms between the ecotypes. This study provides information on the HS response of chickens, adapted to two different agro climatic environments, extending our understanding of the mechanisms of HS response and the effect of adaptation in counteracting HS.

## Introduction

Chicken is a cheap source of high-quality protein and provides significant food and income security for rural communities (Mekonnen et al., 2010). Specialized trait selection, under controlled environments, has made commercial broiler chickens sensitive to environmental extremes (Mcmichael et al., 2007; Ciscar et al., 2011; Kantanen et al., 2015). This creates significant hindrance for the expansion of poultry industry into regions that experience environmental conditions such as heat stress (HS) (Canario et al., 2013; Lawrence and Wall, 2014; Rothschild and Plastow, 2014). Being homeothermic, chickens are able to maintain a constant body temperature across a wide range of temperature (Deeb and Cahaner, 1999); however, increasing ambient temperature due to global warming and climate change will have a major impact on the animal’s physiology and performance, resulting in significant economic losses to livestock industries (Renaudeau et al., 2012; Wang et al., 2017). HS is classified as the state at which the ambient temperature exceeds the tolerable range, making it difficult for the birds to maintain its homeostatic body temperature (Lara and Rostagno, 2013). It leads to reduction in meat quality, low growth rate, reductions in body weight, reduced egg weight and shell thickness, and also high mortality in commercial layers and boilers (Muiruri and Harrison, 1991; Wolfenson et al., 2001). They also cause significant immunosuppression due to reduced humoral immunity, rendering the birds susceptible to diseases (Padgett and Glaser, 2003; Wang et al., 2017; Monson et al., 2018). The birds’ response to HS depends on its genetics (Felver-Gant et al., 2012). While commercial fast-growing broilers are particularly more sensitive to HS (Yunis and Cahaner, 1999), indigenous chicken (IC) breeds that are native to tropical areas are documented to have higher HS tolerance relative to other breeds (Soleimani and Zulkifli, 2010), suggesting that genetic resistance to HS can be acquired as a consequence of adaptation and can be inherited (Lu et al., 2007).

Future agricultural expansion, to support increasing global population, will mainly happen in regions with climatic conditions that are less suitable for commercial livestock, which lack the genetic potential to adapt to environmental extremes (Lara and Rostagno, 2013; Porto-Neto et al., 2014; Rothschild and Plastow, 2014). In the villages of developing countries like Kenya, IC production provides not only food security but also income security due to their low production cost and their ability to survive on scavenging and their resilience to environmental parasite challenge (Magothe et al., 2012). It was reported that 70% out of a total of 31.8 million domesticated chickens in Kenya were IC (Moraa et al., 2015). Kenya has seven different agroecological zones, including, arid, semi-arid, tropical, and temperate regions (Silvestri et al., 2012). Twelve ecotypes of chickens are found across these agroecological zones (Kingori et al., 2010; Moraa et al., 2015). The chickens show high genetic diversity and are well adapted to their local environment (Nyaga, 2007). The chickens are raised extensively under free range systems (Sonaiya, 1990), which exposes them to the negative influence of extreme weather changes. Native chicken ecotypes that have survived extreme environmental conditions over multiple generations would have developed tolerance at the genomic level (Chen et al., 2014; Lawrence and Wall, 2014; Porto-Neto et al., 2014; Fleming et al., 2017). Therefore, to mitigate impacts of HS through genetic approaches, it is prudent to examine chickens that have evolved in such environments (Fleming et al., 2017). Kenyan IC presents an opportunity to understand the genetic response to HS of hot temperature–adapted chickens. Previous studies on HS adapted and nonadapted chickens revealed the biological mechanisms regulated by HS and identified differential immune response between lowland- and highland-adapted chickens exposed to tropical conditions (Park et al., 2019; Te Pas et al., 2019). In this study, we exposed chickens collected from local farmers in Mombasa, which is located at an elevation of approximately 50 m (lowland) in the Kenyan coast with an average temperature between 22°C and 35°C (Njarui et al., 2016), and from Naivasha, located at an elevation of approximately 1800 m (highland) with an average temperature of 8°C to 26°C (Ouko et al., 2017), to a short-term HS treatment (acute) and a repeated longer-term HS treatment (chronic) and analyzed the transcriptome response of skeletal and cardiac tissues using RNA sequencing. Exposure of chicken embryo to elevated temperature induces an adaptive response to HS at later stages in their life (Janke et al., 2004; Loyau et al., 2014; Loyau et al., 2015; Loyau et al., 2016; Fleming et al., 2017). It was hypothesized that chickens that were hatched at relatively higher temperature in the lowlands would respond to HS differently than the highland chickens that were hatched and raised at a lower temperature. Comparative transcriptome analysis by measuring global gene expression changes between the two will identify important genes and pathways that are critical for response to HS. We performed pairwise differential gene expression analysis between control and treatment groups at each time point and identified functional difference in response to HS between the tissues of the two chicken types. We then performed gene coexpression network (GCN) analysis by integrating the different differentially expressed gene (DEG) datasets generated from lowland and highland chickens to understand the overall response of the two ecotypes to HS.

## Materials and Methods

### Experimental Design

**Figure 1:**
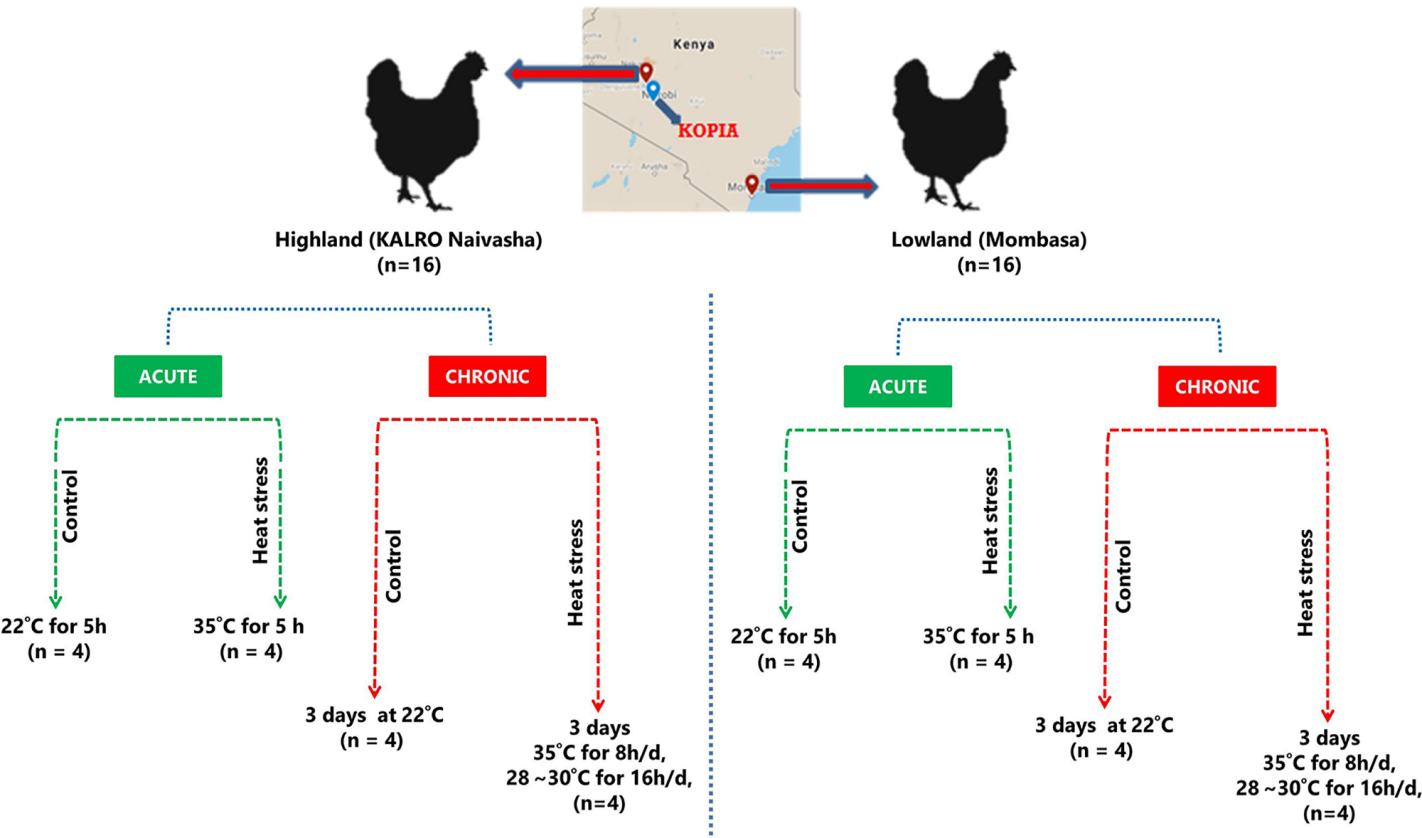
Schematic experimental design.

The study involved two groups of chickens; one was collected from the lowland (low altitude) region and another from the highland (high altitude) regions of Kenya. The lowland chickens were collected from local farmers in Mombasa (4°1′0″S, 39°35′24″E) (average temperature between 22°C and 35°C), while the highland chickens were obtained from KALRO (Kenyan Agricultural and Livestock Research Organization) in Naivasha (average temperature between 8°C and 26°C). A schematic of the experimental design is given in **Figure 1**. A total of 32 (n = 16 from each region), 5-month-old female chickens were used in this study. The HS experiments were conducted at the KOPIA (Korea Project for International Agriculture) Kenya center at Nairobi. The birds had *ad libitum* access to feed and water. After acclimating the birds to the local environment in the experimental cage for 3 days, the experiments were performed. The experiments were performed in a specially designed cage fitted with a temperature controller (**Supplementary Figure 1**). The HS group (n = 16) was exposed to high temperatures of 35°C for 8 h per day (9:00–17:00 h) and remained at 28°C to 30°C at all other times. The control group (n = 16) was maintained at 24°C during the entire experimental period. The short-term HS group (acute group) (n = 16, four per region, including the controls) were euthanized after 5 h of increased temperature exposure, and cardiac and skeletal muscle tissues were collected. The long-term HS group (C) (n = 16, four per ecotype, including the controls) were euthanized at the end of 3 days of cyclic HS, and cardiac and skeletal muscle tissues were collected. Rectal temperatures were measured at the beginning and end of the treatment period using a temperature probe. A total of 64 samples were collected; they were stored in RNAlater (Ambion, Texas, USA) and transported to the National Institute of Animal Science (South Korea) and stored at −80°C until further use.

### RNA-Seq Analysis

Total RNA was isolated from 32 skeletal muscles and 32 cardiac muscles with RNeasy mini kit (Qiagen, USA) following the manufacturer’s protocol. The purity and concentration of the isolated RNA were measured with NanoDrop ND-1000 UV-vis Spectrophotometer (NanoDrop Technologies Inc., Wilmington, DE, USA). The integrity of the RNA was measured on Bioanalyzer 2100 system using RNA Nano 6000 Assay kit (Agilent Technologies, CA, USA), and only samples with a RIN (RNA integrity number) value greater than 8 were used for sequencing. cDNA libraries were generated using Illumina TruSeq^®^ RNA sample preparation v2 kit (Illumina, San Diego, CA, USA) following processes previously described (Srikanth et al., 2017b). Quality of the individual libraries was accessed on Bioanalyzer 2100 system using DNA Nano 1000 Assay kit. Paired-end (PE) sequencing was performed on Illumina^®^ HiSeq 2000 on four lanes (21, 12, 7, and 24 samples on lanes 1, 4, 5, and 6, respectively) of a single chip, blocking by treatment, tissue, and region. The sequencing was carried out by Macrogen (Seoul, South Korea). The raw reads are freely available at the NCBI (National Center for Biotechnology Information) SRA (Sequence Read Archive) database under accession number PRJNA557270. The quality of the raw reads was accessed using FastQC (version 0.11.5) (Andrews, 2010). Reads shorter than 80 base pairs (bp), low-quality bases, and adapters were removed using TRIMMOMATIC (version 0.36) (Bolger et al., 2014). All 100-bp reads were individually aligned to the chicken reference genome (*Gallus gallus* 5.0, release 94, Ensembl) using HISAT2 (version 2.0.5) (Kim et al., 2016) following methods previously described (Park et al., 2019). The DEGs were identified using CUFFLINKS (version 2.2.1) (Trapnell et al., 2012). The -G/–GTF flags was used to quantitate against reference transcript annotations. The expression of individual genes were measured as fragments per kilobase of exon per million, and DEGs (FDR < 0.05) were identified with CUFFDIFF. Eight DEG gene sets were generated (highland: control-acute, control-HS, HS-acute, HS-chronic; lowland: control-acute, control-HS, HS-acute, HS-chronic). Functional annotation and overrepresentative analyses were carried out using the web-based gene ontology (GO) clustering tool DAVID (Huang et al., 2008). The genes were annotated under the Biological Process, Molecular Function, and KEGG pathway terms. Significant terms (FDR <0.05 for KEGG and FDR <0.05 for GO terms) were plotted with ggplot2 package in R (version 3.4.1) (Team, 2013). The Venn diagrams were generated with a web-based tool (http://bioinformatics.psb.ugent.be/webtools/Venn/). 

### GCN Analysis and KEGG Pathway Mapping

GCN was constructed using the partial correlation coefficient with information theory (PCIT) algorithm (Reverter and Chan, 2008). We constructed two networks (a lowland-specific network and a highland-specific network), using DEGs (differentially expressed), in at least one of the four gene sets that were generated from the lowland or highland chicken groups. Only genes that had a partial correlation |*r*| of ≥0.99 were included for network construction. The networks were visualized in CYTOSCAPE (version 3.4.1) (Shannon et al., 2003), analyzed with the NetworkAnalyzer plugin, and sorted according to degrees of connections. The genes in the network were then mapped to Kyoto Encylopedia of Genes and Genomes (KEGG) pathways (Ogata et al., 1999; Kanehisa and Goto, 2000) using the ClueGO plugin (Bindea et al., 2009).

### Quantitative Reverse Transcriptase–Polymerase Chain Reaction Analysis

One microgram of each of the isolated RNA was reverse transcribed into cDNA with Oligo(dT) using SuperScript III^™ ^first-strand system for reverse transcriptase–polymerase chain reaction (RT-PCR) (Invitrogen, CA, USA) in a final volume of 20 μl using the manufacturer’s protocol. The resulting cDNAs were diluted 1:2, prior to their use for quantitative RT (qRT)–PCR analysis. The PCR reactions were carried out at a final volume of 10 μl containing 5 μl of Universal Master Mix containing dNTPs. MgCl_2_, reaction buffer and AmpliTaq Gold DNA polymerase, 90 nM of primers (forward and reverse) and 250 nM of fluorescence-labeled TaqMan probe, and finally 2 μl of the cDNA. Amplifications were carried out on an ABI PRISM 7900HT Sequence Detection Systems (Applied Biosystems, CA, USA) with initial denaturing for 10 min at 95°C, followed by 40 cycles of 95°C for 15 s and 60°C for 1 min. All samples were amplified in triplicates. The data were analyzed with the SEQUENCE DETECTOR software (Applied Biosystems). All reagents used in the qRT-PCR analysis were procured from Life Technologies (Carlsbad, CA, USA). The absolute fold change was calculated after normalization with the chicken glyceraldehyde-3-phosphate dehydrogenase gene (GAPDH), using the 2^-ΔΔCT^ method (Schmittgen and Livak, 2008). All the primers used in the analyses are listed in **Table 1**.

**Table 1:** List of primers used for qRT-PCR validation of RNA-Seq results.

Gene	Primer sequencing (5′ –> 3′)
HSPH1	F –> TGAATTGGAAACTCAGGACCAGATG
	R –> CCTCACTGTTCTCTTGCTGGTTATT
SRGN	F –> GGCCGTGGTTCCAGCT
	R –> GCTCCTGGTACGTCTTCATCAG
ATRAID	F –> GTTGGACCTCAGCAACTGTTC
	R –> CGTCAGGTCCAGCACGAC
MT4	F –> CGGAGCTGCCGCAAGA
	R –> CCTTGGCACAGTTGTTGCA
PDK4	F –> TGACTGGTCGCATCCCAAGTAAG
	R –> GGAAGAATTTGCCTGTTTGGAGG
OTUD1	F –> GCTGTGTCCCTCTCCAAGATG
	R –> CACGCTTCTGTCCGTCTGT
GAPDH	F –> GGAAGAATTTGCCTGTTTGGAGG
	R –> TCGTCAAGCTTGTTTCCTGGTATGA

## Results

All the chickens (n = 32) procured from the lowland Mombasa region and the highland Naivasha region were brought to the KOPIA center in Nairobi. After acclimating the birds to the local environment and the cage, the lowland and highland birds were randomly separated into four groups each (n = 4/group), comprising the acute stage groups: ALL (acute and lowland) and AHL (acute and highland); and the chronic stage groups; CLL (chronic and lowland) and CHL (chronic and highland). Each of these stages comprised a treatment group and a control groups (**Figure 1**).

### Effect of HS on Rectal Temperature

**Figure 2:**
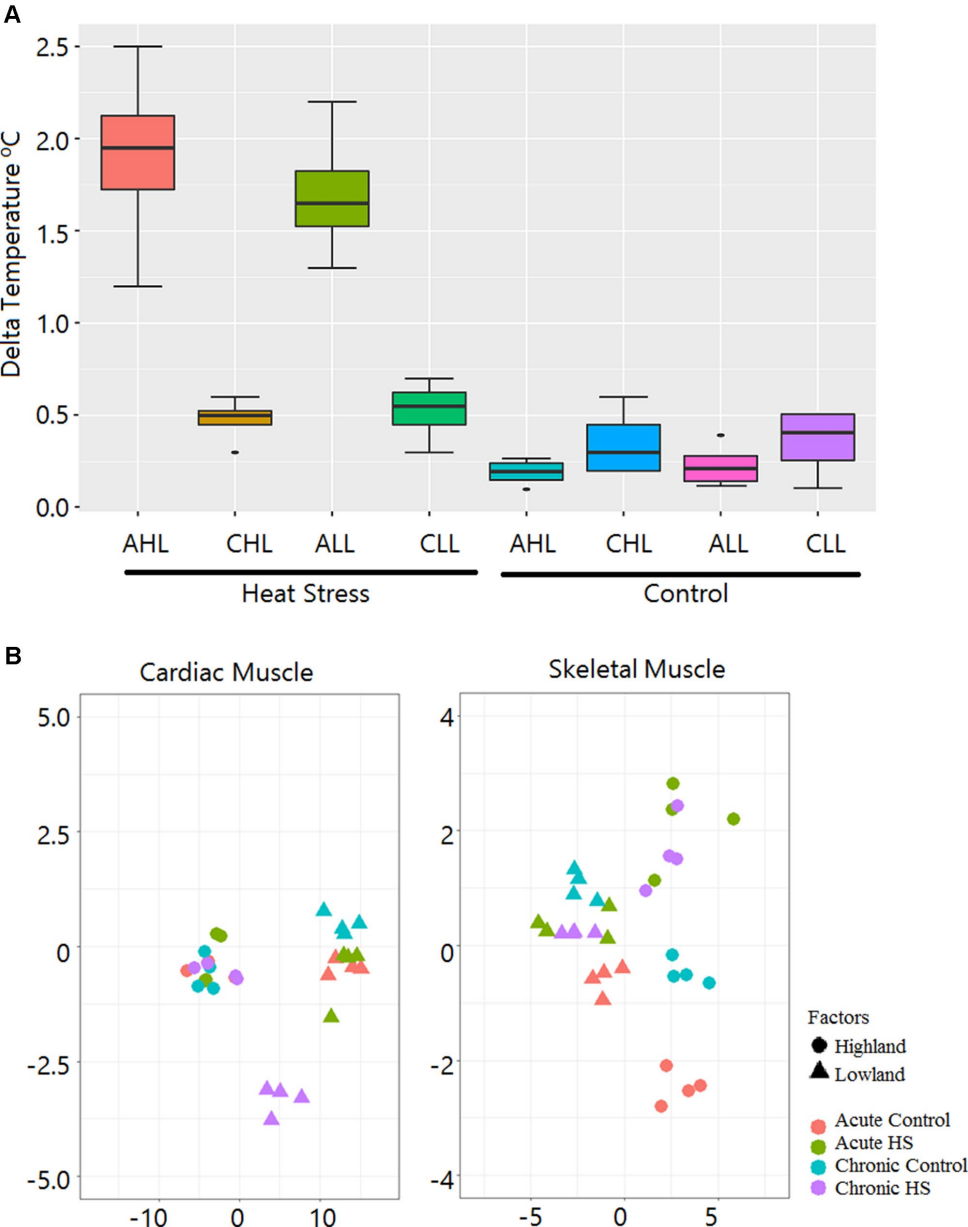
Rectal temperature gain during HS treatment and sample relationship inferred from principal components analysis. **(A)** Box plot showing gain in rectal temperature due to HS treatment. AHL, acute highland; CHL, chronic highland; ALL, acute lowland; CLL, chronic lowland. **(B)** Principal components analysis showing that the maximum variation is due to differences between the ecotypes. Only a small percentage of the variation is due to the HS effect.

**Figure 2A** shows the changes in the rectal temperatures of the animals before the start and at the end of the experiment. The maximum increases in rectal temperatures were in the AHL and the ALL HS groups. On an average, the rectal temperatures of AHL and ALL birds increased by 1.8°C and 1.6°C, respectively. Only minor changes in temperature were noted in the control groups.

### Transcriptome Alignment and Mapping Statistics

We constructed 64 cDNA libraries from the cardiac and skeletal muscle tissues of the lowland and highland Kenyan chicken groups from the two experimental time points (acute and chronic). There were 1.33 billion (647 million in cardiac and 650 million in skeletal muscle), 100-bp PE reads corresponding to an average of 1.65 Gb of sequence data per sample that were generated. After trimming for adapters and low-quality reads, 1.29 billion reads corresponding to 97.56% of total sequenced reads were used for downstream analysis. The reads were mapped to the chicken genome at an average alignment rate of 91.1% and 85.12% for cardiac and skeletal muscle tissues, respectively. A summary of the mapping statistics is given in **Supplementary File 3**. Principal components analysis showed that the maximum variation is due to difference between the ecotypes, and only a small percentage of the variation is due to HS effect (**Figure 2B**). A list of all the DEGs identified in this study is given in **Supplementary File 1**.

### Effects of Acute HS on the Cardiac and Skeletal Muscle Transcriptome of the Highland and Lowland Chickens

**Figure 3:**
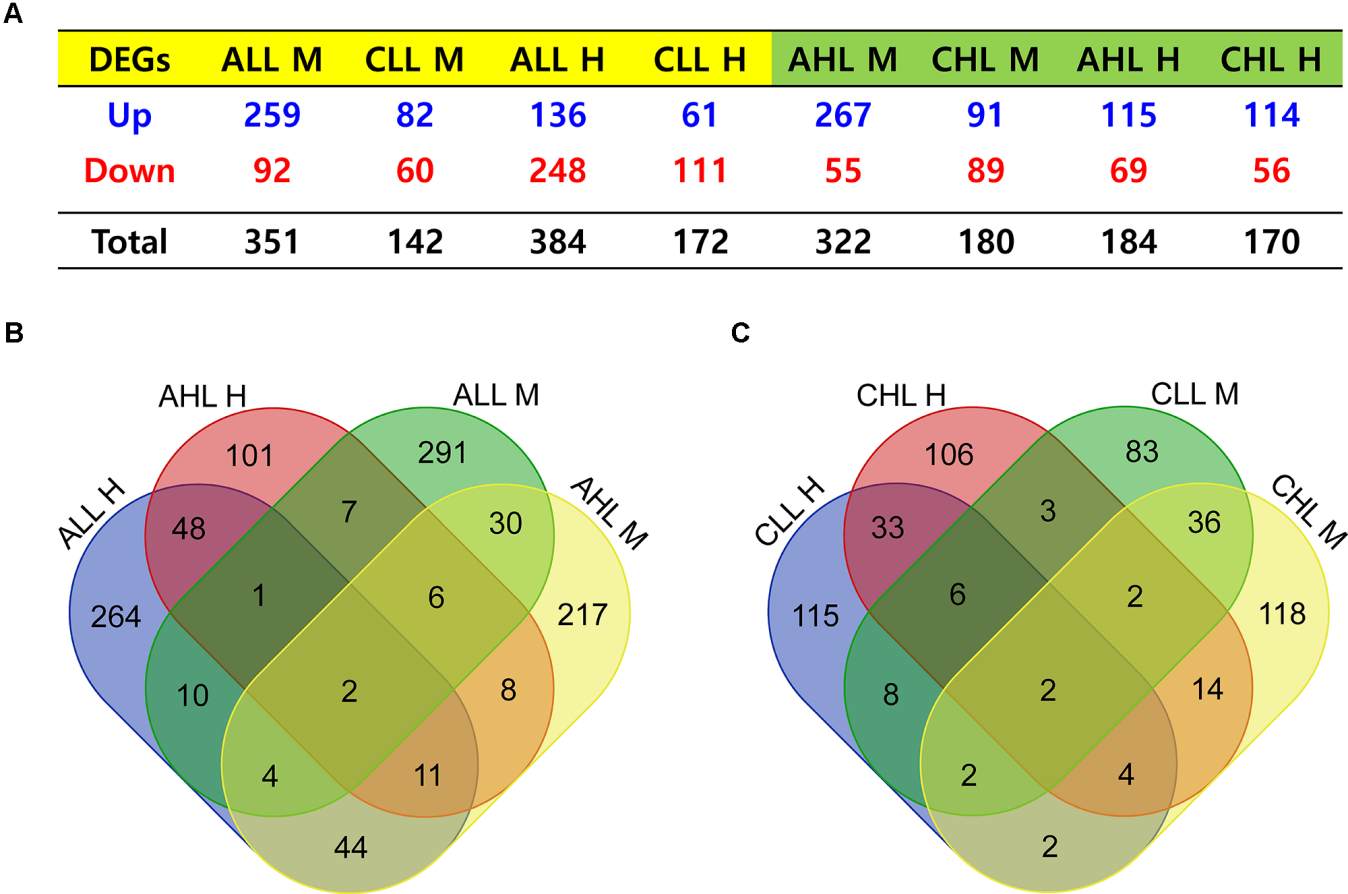
Genes differentially expressed relative to control group under different contrast. **(A)** Number of genes that were up-regulated or down-regulated after HS treatment. **(B)** Venn diagram showing common and unique genes differentially expressed during the acute stage treatment. **(C)** Venn diagram showing common and unique genes differentially expressed during the chronic stage treatment. M and H, skeletal muscle and cardiac muscle, respectively; AHL, acute highland; CHL, chronic highland; ALL, acute lowland; CLL, chronic lowland.

**Figure 4:**
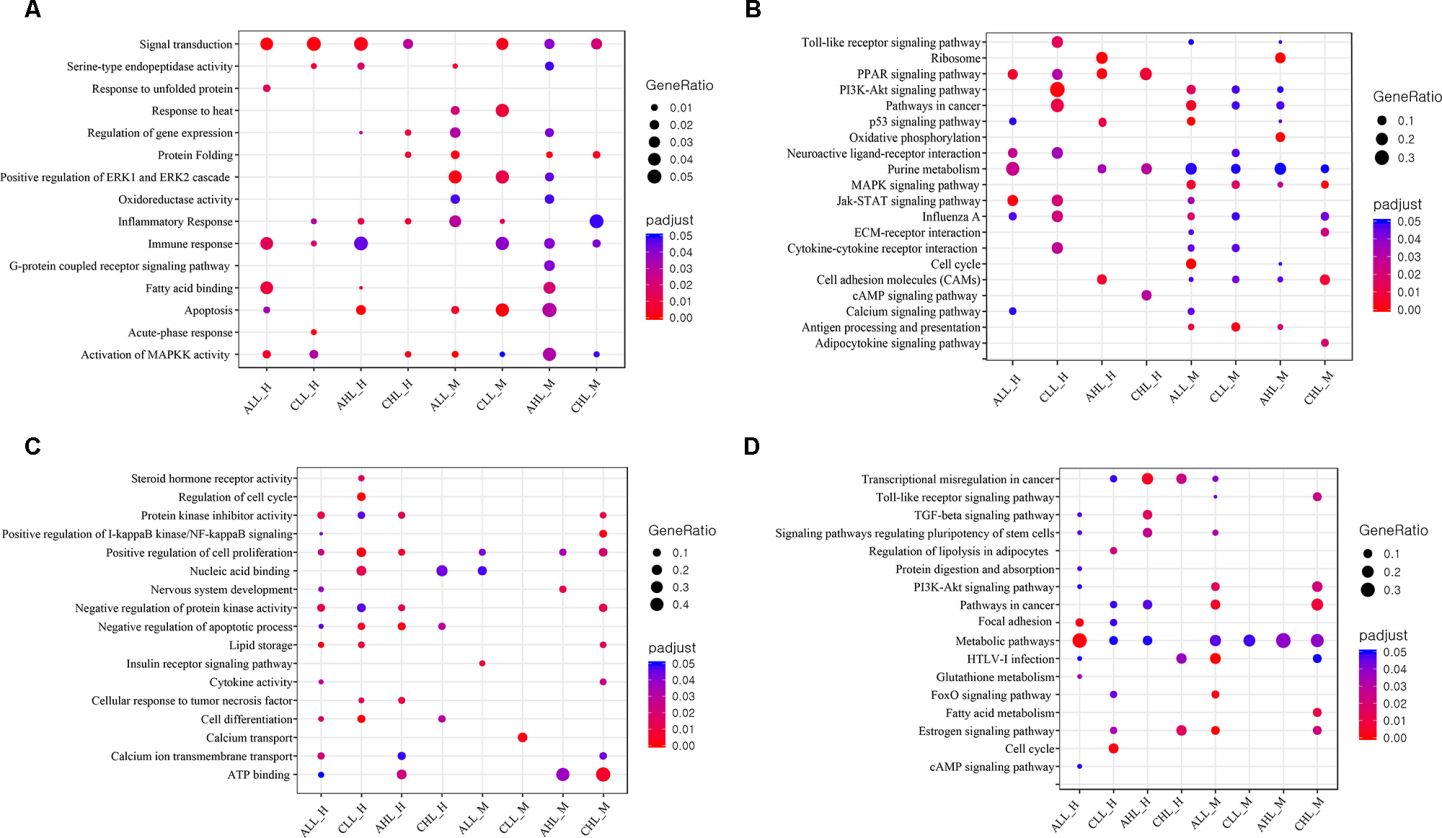
GO and KEGG pathway enrichment analysis **(A)** Dot plot shows the up-regulated GO terms (FDR <0.05) of biological processes and molecular functions identified using DAVID to be enriched under the different contrasts. **(B)** Dot plot shows the up-regulated KEGG pathways (FDR <0.1) enriched for different contrasts. The size of the dot is based on gene count enriched in the pathway, and the color of the dot shows the pathway enrichment significance. **(C)** Dot plot shows the down-regulated GO terms (FDR <0.05) of biological processes and molecular functions identified using DAVID to be enriched under the different contrasts. **(D)** Dot plot shows the down-regulated KEGG pathways (FDR <0.1) enriched for different contrasts. The size of the dot is based on gene count enriched in the pathway, and the color of the dot shows the pathway enrichment significance.

Acute HS resulted in 351 and 322 genes in the skeletal muscle and 384 and 184 DEG in the cardiac tissues to be significantly differentially expressed (FDR <0.05) in the lowland and the highland, respectively (**Figure 3A**). Between the two ecotypes, 48 and 30 DEGs overlapped between the cardiac and skeletal muscle tissues, respectively (**Figure 3B**). Ten DEGs were commonly differentially expressed between the two tissues in the lowland chickens; eight DEGs differed between the two tissues in the highland chickens (**Figure 3B**). Only two DEGs were found in all four contrasts. These were heat shock protein (HSP) family A (Hsp70) member 8 (HSPA8) and HSP family B (small) member 7 (HSPB7). GO enrichment analysis (**Figure 4A**) of the up-regulated DEGs showed that at the acute stage in the cardiac tissue of lowland chicken, the most significant terms (*Q* <0.05) enriched were “signal transduction,” “immune response “activation of MAPKK activity,” “fatty acid binding,” “response to unfolded protein,” and “apoptosis,” while in the highland chickens “signal transduction,” “serine-type endopeptidase activity,” “inflammatory response,” “immune response,” and “apoptosis” were the most significantly enriched terms. In the skeletal muscle of lowland chickens, the GO terms enriched were “response to heat,” “protein folding,” “apoptosis,” “oxido-reductase activity,” “activation of MAPKK activity,” and “positive regulation of ERK1 and ERK2 cascade,” while in the highland chickens, “signal transduction,” “protein folding,” “apoptosis,” “oxido-reductase activity,” “activation of MAPKK activity,” and “immune response.” Among the down-regulated DEGs (**Figure 4C**) “protein kinase inhibitor activity,” “positive regulation of cell proliferation,” “negative regulation of apoptotic process,” lipid storage,” and “cell differentiation” were significantly down-regulated in cardiac tissues of acute stage lowland chickens, while “positive regulation of cell proliferation,” “negative regulation of apoptotic process,” “cellular response to tumor necrosis factor,” and “ATP binding” were down-regulated in the highland chicken. In the skeletal muscle, “insulin receptor signaling” and “calcium transport” were the most significantly enriched terms in the acute stage in the lowland chickens, while “ATP binding,” “nervous system development,” and “positive regulation of cell proliferation” were significantly enriched in the highland chickens. The enrichment analysis showed that several biological processes were regulated in more than one situation.

KEGG pathway enrichment analysis of up-regulated DEGs (**Figure 4B**) showed that in the cardiac muscles of the lowland chickens the most enriched pathways (FDR <0.05) were “Jak-STAT signaling pathway,” “PPAR signaling pathway,” “purine metabolism,” “neuroactive ligand–receptor interaction,” and “p53 signaling,” while in the skeletal muscle tissue “cell cycle,” “MAPK signaling pathway,” “pathways in cancer,” “antigen processing and presentation,” and “p53 signaling pathway” were enriched. In the cardiac tissues of highland chickens, “PPAR signaling pathway,” “cell adhesion molecules,” and “p53 signaling pathway” and in the skeletal muscle “MAPK signaling,” “oxidative phosphorylation,” “PPAR signaling pathway,” “ribosome,” and “purine metabolism” were enriched. Among the down-regulated DEGs (**Figure 4D**) in the cardiac tissue of lowland chickens, “metabolic pathways” and “focal adhesion” were significantly down-regulated, while “estrogen signaling pathway,” “HTLV-I infection,” and “FoxO signaling pathway” were down-regulated in skeletal muscle. In the cardiac muscle of highland chickens, “transcriptional misregulation in cancer” and “TGF-beta signaling pathway” were significantly down-regulated, while “metabolic pathways” was down-regulated in the skeletal muscle.

### Effects of Chronic HS on the Cardiac and Skeletal Muscle Transcriptome of the Highland and Lowland Chickens

Under chronic HS, 142 and 172 DEGs were found in the skeletal and cardiac tissues of the lowland chickens, while 180 and 170 DEGs were found in the skeletal and cardiac tissues of the highland chickens (**Figure 3A**). Between the two chicken ecotypes, 33 DEGs were common between the cardiac and 36 DEGs were common between the skeletal muscle tissues (**Figure 3C**). Eight DEGs were common between the two tissues in the lowland chicken; 14 DEGs were common between the two tissues in the highland chicken (**Figure 3C**). Only two DEGs were common in all the four contrasts; these were the HSP family A (HSP70) member 8 (HSPA8) and fatty acid–binding protein 4 (FABP4) (**Figure 3C**), these two genes were previously found be differentially expressed in the hypothalamus of a meat-type chicken (Sun et al., 2015a). GO enrichment analysis of the up-regulated DEGs (**Figure 4A**) showed that in the lowland chickens “signal transduction,” “acute-phase response,” “immune response,” “inflammatory response,” and “serine-type endopeptidase activity” were enriched in the cardiac tissue, while “apoptosis,” “response to heat,” “positive regulation of ERK1 and ERK2 cascade,” and “immune response” were enriched in skeletal muscle. In the highland chicken, “regulation of gene expression,” “protein folding,” “inflammatory response,” and activation of MAPKK activity” were enriched in the cardiac tissues, while “inflammatory response,” “protein folding,” and “signal transduction” were enriched in skeletal muscle tissues. Among the down-regulated DEGs (**Figure 4C**), “regulation of cell cycle,” “positive regulation of cell proliferation,” “nucleic acid binding,” “lipid storage,” and “cell differentiation” were enriched in the cardiac muscle of lowland chickens, while “calcium transport” was enriched in the skeletal muscle. In the highland chicken, “negative regulation of apoptotic process,” “cell differentiation,” and “nucleic acid binding” were enriched in the cardiac muscle, while “ATP binding,” “positive regulation of I-kappaB kinase/NF-kappa signaling,” and “protein kinase inhibitor” were enriched in the skeletal muscle.

KEGG pathway enrichment analysis of up-regulated DEGs (**Figure 4B**) showed that in lowland chickens “PI3K-Akt signaling,” “pathways in cancer,” “Jak-STAT signaling,” and “Toll-like receptor signaling pathway” were enriched in the cardiac tissue, and “MAPK signaling pathway,” “antigen processing and presentation,” and “cell adhesion molecules” were enriched in the skeletal muscle. In the highland chickens, “PPAR signaling pathway,” “purine metabolism,” and “cAMP signaling” pathways were enriched in the cardiac and “MAPK signaling pathway,” “ECM–receptor interaction,” and “cell adhesion molecules” pathways were enriched in the skeletal muscle. “Cell cycle,” “regulation of lipolysis in adipocytes,” and “estrogen signaling” pathways were down-regulated (**Figure 4D**) in the cardiac muscle, while “metabolic pathway” was down-regulated in the skeletal muscle in lowland chicken. In the highland chicken, “estrogen signaling pathway,” “HTVL-I infection,” and “transcriptional misregulation in cancer” were down-regulated (**Figure 4D**) in cardiac muscle, while “pathways in cancer,” “fatty acid metabolism,” and “metabolic” pathways were down-regulated in the skeletal muscle.

### Integration of Cardiac and Skeletal Muscle DEGs to Understand the Overall Response of the Highland and Lowland Chickens to HS

**Figure 5:**
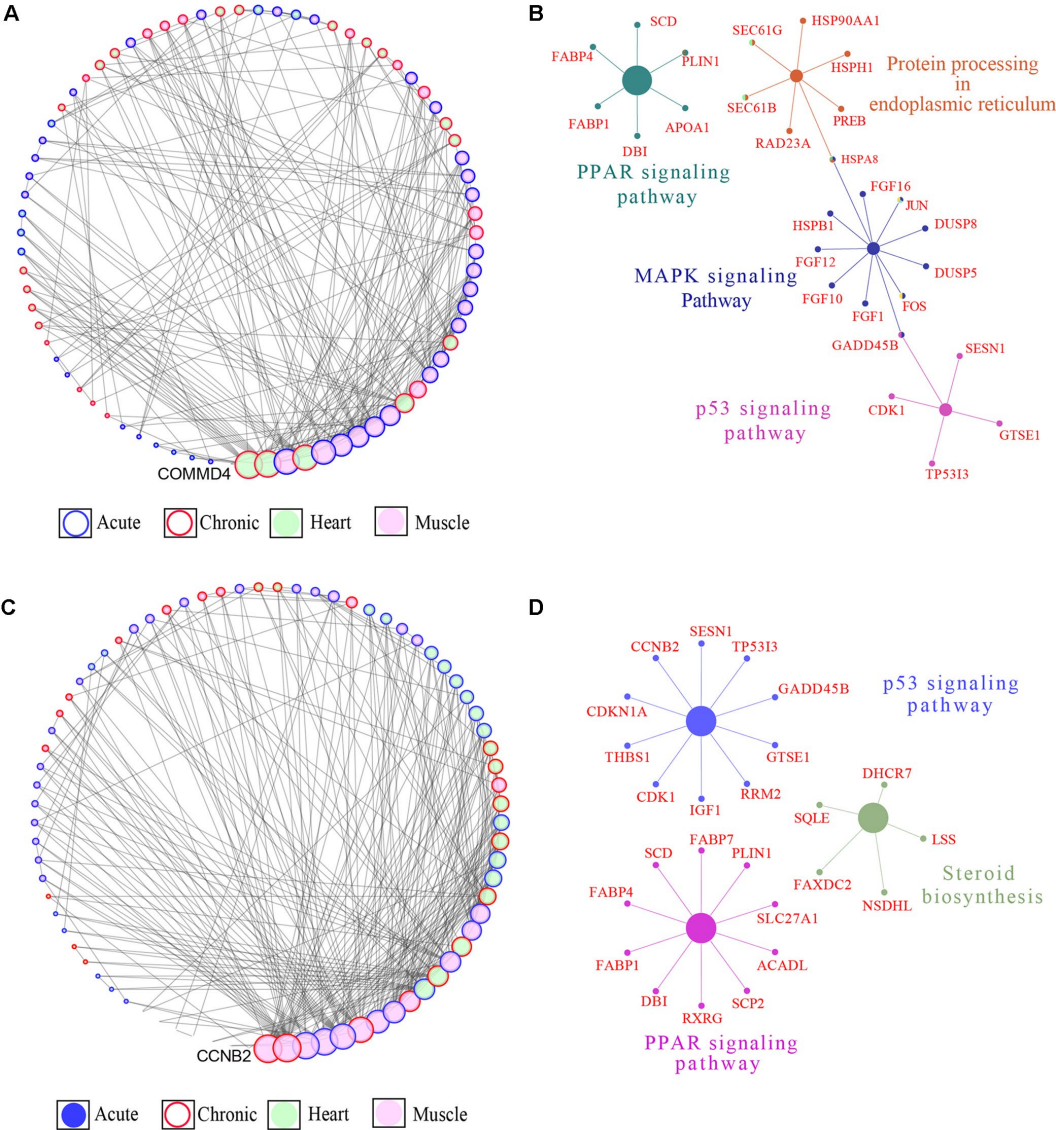
Gene coexpression network (GCN) and pathway enrichment analysis integrated for the skeletal and cardiac muscle DEGs. **(A)** Degree sorted network of DEGs in at least one contrast in the highland chickens. The nodes are genes, and the edges are based on correlation coefficients. Only genes with a partial correlation | *r* | of ≥0.99 were included in network. Node color denotes the tissue type in which the gene expression was the highest, while node border denotes the stage at which the gene expression was the highest. **(B)** KEGG pathway networks in which all the genes in the highland GCN network were enriched. **(C)** Degree sorted network of DEG in at least one contrast in the lowland chickens. The nodes are genes, and the edges are based on correlation coefficients. Only genes with a partial correlation | *r* | of ≥0.99 were included in network. Node color denotes the tissue type in which the gene expression was the highest, while node border denotes the stage at which the gene expression was the highest. **(D)** KEGG pathway networks in which all the genes in the lowland GCN network were enriched.

We integrated the DEGs identified in the acute and chronic stages in the cardiac and skeletal muscle tissues of the highland (**Figure 5A**) and lowland (**Figure 5C**) chickens through a GCN constructed based on PCIT (Reverter and Chan, 2008). The highland GCN comprised of 75 nodes (genes) and 244 edges (connections) (**Figure 5A**). KEGG pathway enrichment analysis of the genes in the network showed that four pathways comprising 28 of the 77 genes in the networks were enriched; these included “PPAR signaling pathway,” “protein processing in endoplasmic reticulum,” “MAPK signaling pathway,” and “p53 signaling pathway’ (**Figure 5B**). The lowland gene network comprised 77 nodes (genes) and 270 edges (connections) (**Figure 5C**). Three KEGG pathways comprising 25 of the 75 genes in the network were found to be enriched; these included “p53 signaling,” “steroid biosynthesis,” and “PPAR signaling” pathways (**Figure 5D**). The networks were sorted according to degree (number of edges incident to the node [genes]). A list of all the genes in the network and their degree is given in **Supplementary File 2**. Genes with the maximum connections (degrees) in the lowland network included cyclin-B2 (*CCNB2*), crumbs homolog 2 (*Crb2*), carbohydrate sulfotransferease 9 (*CHST9*), sestrin-1 (*SESN1*), and nuclear receptor subfamily 4 group A member 3 (*NR4A3*), while in the highland coexpression network COMM domain containing 4 (*COMMD4*), tetratricopeptide repeat domain containing protein 32 (*TTC32*), H1 histone family member 0 (*H1F0*), acylphosphatase 1 (*ACYP1*), and ribosomal protein S28 (*RPS28*) had the highest degree. Comparison of the two networks revealed that 30 genes were shared between the two networks (**Supplementary File 2**); among these were genes involved in PPAR signaling pathway (*PLIN1*, *SCD*, *FABP4*, *FABP1*, and *DBI*), p53 signaling pathway (*CDK1*, *TP53I3*, *GADD45B*, *SESN1*, *GTSE1*), and MAP Kinase signaling pathway (*DUSP5* and *DUSP8*).

### Validation of RNA-Seq Results

**Figure 6:**
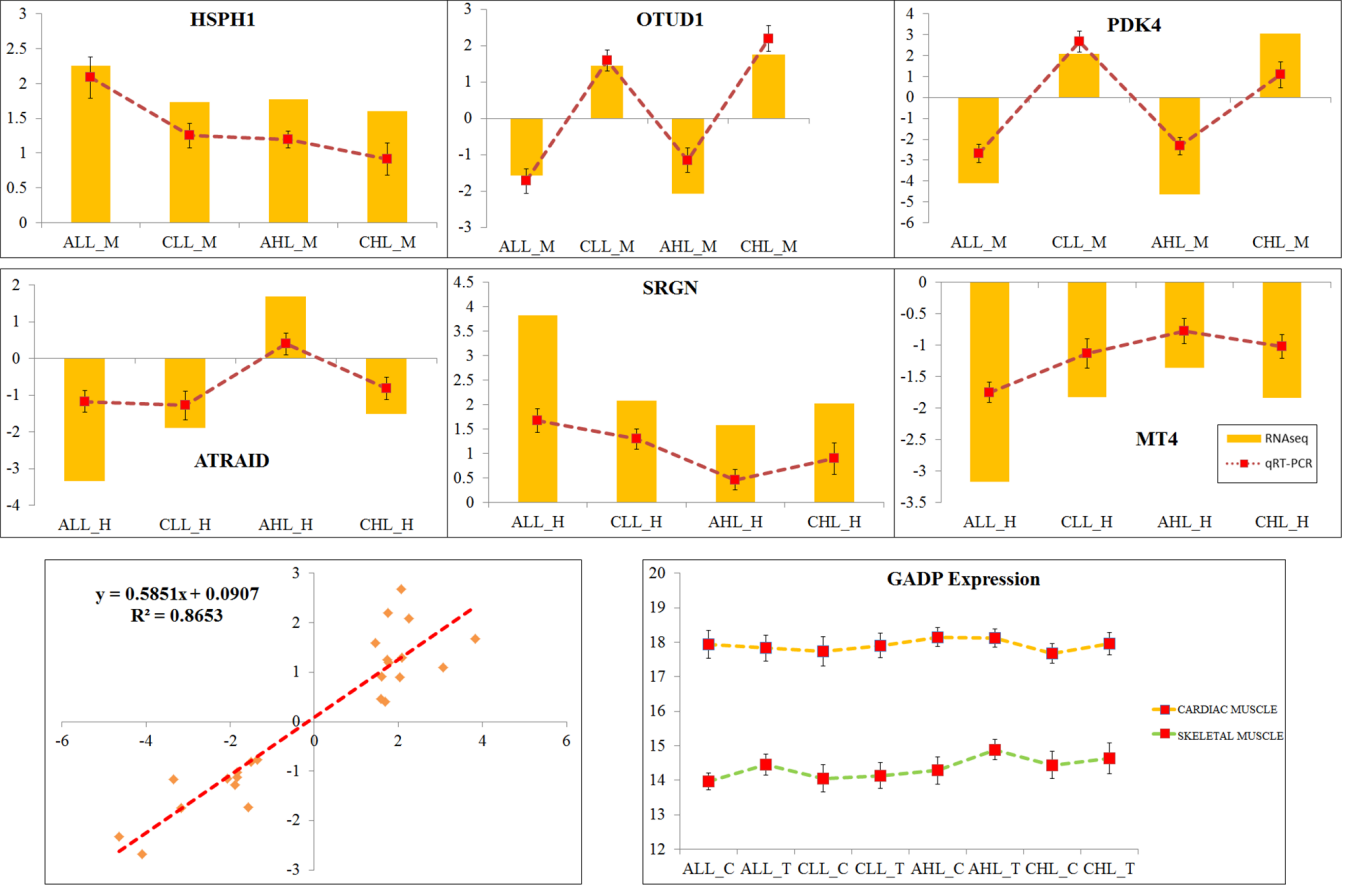
Validation of RNA sequencing results with qRT-PCR analysis. The real-time PCR analyses of HSPH1, OTUD1, PDK4, ATRAID, SRGN, and MT4 were performed on all the samples that were used in the RNA-Seq analysis. GADPH, which showed constant expression in all samples, was used as a normalization gene. Log2 fold changes identified in the RNA-Seq analysis were plotted as bar, and the fold changes through qRT-PCR were plotted as line graph. A scatter plot of RNA-Seq Log2 fold change and the qRT-PCR fold change were plotted, and the correlation (*r* ^2^) between the two methods was identified.

Out of 30 genes that overlapped between the two coexpression networks, six genes were randomly chosen for validation by qRT-PCR analysis. **Figure 6** shows the PCR quantification of the *OTUD1*, *HSPH1*, *PDK4*, *ATRAID*, *SRGN*, and *MT4* genes. The results broadly showed a similar expression profile between the RNA-Seq and qRT-PCR. A correlation of 0.86 was observed between the RNA-Seq and qRT-PCR log2 fold-change results (**Figure 6**).

## Discussion

High ambient temperatures affect the production and reproduction rates in animals (Srikanth et al., 2017a). Studies have highlighted the deleterious effect of HS on physiological (Altan et al., 2003; Mujahid et al., 2007), biochemical (Xie et al., 2015), and immune capacity of chickens (Park et al., 2019; Te Pas et al., 2019) (Altan et al., 2003; Mujahid et al., 2006; Mujahid et al., 2007; Huang et al., 2015; Xie et al., 2015; Park et al., 2019; Te Pas et al., 2019). Fast-growing, commercial chickens, artificially selected and raised under controlled environment, are very sensitive to HS and might not have the genetic potential to develop thermal tolerance, limiting their potential for rearing in developing countries (Coble et al., 2014; Lan et al., 2016; Fleming et al., 2017). Native breeds and village ecotypes that have been under environmental challenges such as high ambient temperature over multiple generations might have developed thermal tolerance due to adaptation to local conditions (Clarke, 2003; Chen et al., 2009; Seebacher, 2009; Nardone et al., 2010; Lawrence and Wall, 2014; Porto-Neto et al., 2014); examining such native breeds will provide us with genetic information needed to mitigate the impact of HS. In this study, we explored the transcriptomic response of Kenyan chicken ecotypes collected from two different environmental regions. The cardiac muscle was chosen due to its central role in heat dissipation through blood circulation (Zhang et al., 2017), and skeletal muscle was chosen due to its susceptibility to HS-induced oxidative damage and damages to membrane integrity (Sandercock et al., 2001; Mujahid et al., 2006), which affects meat quality.

### Difference in HS Response Between Acute and Chronic Stages

Considerably higher changes in rectal temperature were noted in the acute group birds, suggesting difficulties in maintaining the core body temperature in response to sudden increase in temperature, compared to the chronic group birds, which were able to regulate their body temperature better at the end of the experimental period. This may be due to acclimation for cyclic HS. The highest change in rectal temperature was observed in the highland chickens of the acute group. These chickens were less adapted to HS as compared to lowland chickens. Therefore, it may be assumed that the response of highland chickens to HS is less robust than the response of lowland chickens. While response to acute HS is under homeostatic regulation (reflex-responsive regulation), the response to chronic HS is under homeorhetic regulation, i.e., metabolic regulation through endocrinal hormones (Collier et al., 2018). Studies have shown that in chickens acute HS, i.e., shock due to sudden change in ambient temperature, is more stressful (Lan et al., 2016) than cyclic HS (Coble et al., 2014). In the lowland chicken, in both cardiac and skeletal muscles, the acute group had 2.2 to 2.5 times more DEGs than the chronic group; however, in the highland chickens, while there were 1.7 times more DEGs in the acute stage of skeletal muscle tissues, a similar number of DEGs were found at both stages in the cardiac tissue (**Figure 3A**). This suggests a difference in heat sensitivity in the cardiac tissues between the lowland and highland chickens. The overall increased number of DEGs in the lowland chickens and the comparatively lesser change in rectal temperature (**Figure 2**) suggest a considerably stronger response to HS than the highland chickens. This difference may denote the difference in acclimatization (rate) between highland and lowland chickens due to a difference in adaptation to HS.

Between the different contrasts in the acute and chronic stages, very few genes overlapped. Overall only two genes in cardiac (HSPA8 and HSPB7) and two genes in skeletal muscle (HSPA8 and FABP4) overlapped. Thus, we conclude that acclimatization leads to a complete change in response to HS. The HSPA8 gene (up-regulated in all contrast) is a member of the HSP70 family of molecular chaperones and is known to play an important role in directing correct folding of newly synthesized proteins and in the destruction of non-reversibly denatured proteins (Hartl, 1996; Iwamoto et al., 2005). HSPA8 has been found to be up-regulated under HS in chickens (Sun et al., 2015b; Wang et al., 2015) and could serve as a good biomarker. HSPB7 is a member of the small HSP (sHSP) family, whose expression is restricted to skeletal and cardiac muscles (Bonomini et al., 2018). HSPB7 functions in protecting cells from protein aggregation (Vos et al., 2010) and is required for maintaining muscle integrity (Juo et al., 2016). HS causes aggregation of denatured proteins (Srikanth et al., 2017a). HSPB7 was found to be significantly up-regulated in lowland chicken’s cardiac (acute and chronic) and skeletal muscle (acute), while the effect was opposite (down-regulated) in the highland chickens. This could indicate that the lowland chickens might be able to counteract the effects of HS-induced protein aggregation better than the highland chickens. We also observed stage-specific difference in HS response (**Figure 4B**). The p53 pathway, which is essential for DNA damage repair, initiation of cell cycle arrest, and cell apoptosis (Rappold et al., 2001; Harris and Levine, 2005), was enriched in both cardiac and skeletal muscles in the acute stage, suggesting that acute HS might have an inhibiting effect on cell cycle; similar observation was noted in liver of heat-stressed broilers (Jastrebski et al., 2017). The PPAR signaling pathway, which is required for energy metabolism (Wang, 2010) and regulating the oxidative stress–induced inflammatory response (Kim et al., 2017), was found to be enriched under chronic HS in three of the four contrasts (**Figure 4B**). Prolonged exposure to heat can cause considerable oxidative stress damage in chickens (Azad et al., 2010; Akbarian et al., 2016). The enrichment of PPAR signaling in the chronic HS group could be indicative of HS-induced reactive oxygen species (ROS) accumulation and oxidative stress. While this may relate to the inflammatory response, it may also just indicate differences between tissues.

### Difference in HS Response Between Cardiac and Skeletal Muscle

The overlapping of very few genes between cardiac and skeletal muscle within and between the two ecotypes (lowland and highland) (**Figures 3B, C**) shows that not only is there a difference in HS response between the tissues, but there is also an ecotype-specific difference in HS response, suggesting differences in HS adaptation. This was indicated by the enrichment of the PPAR signaling pathway in the cardiac and the MAPK signaling pathway in the skeletal muscle. While PPAR signaling pathway regulates energy metabolism and regulation of the oxidative stress response (Wang, 2010; Kim et al., 2017), the MAPK signaling is required for activating programmed cell death (Pearson et al., 2001). The activation of members of the PPAR signaling pathway in cardiac muscle might be due to the requirement of considerable energy for pumping blood to dissipate the accumulated heat or to alleviate oxidative stress, while the enrichment of MAPK signaling genes could be indicative of cellular damage in the skeletal muscle and the triggering of apoptosis.

### Difference in HS Response Between Lowland and Highland Chicken

The DEGs generated under different contrasts were integrated into lowland and highland gene correlation networks (GCNs) to study their overall response to HS. The GCNs were then sorted for number of edges, denoting connections between nodes (genes), to identify hub genes in each ecotype. The top 5 hub genes in the lowland GCN all function in regulating cell cycle, cell signaling, cell division, or in DNA repair mechanism. Cyclin B2 (CCNB2), which is an important regulator of cell mitosis (Brandeis et al., 1998), was significantly up-regulated in lowland skeletal muscle at the chronic stage. CCNB2 was previously found to be significantly down-regulated in the fast-growing ROSS 708 broilers compared to the slow-growing Illinois broiler under HS and was suggested to be indicative of the reduction in cell cycle activity in the Ross broilers (Zhang et al., 2017). The Crb2, a BRCT protein, is a cell cycle checkpoint mediator that is essential for cellular response to DNA damage and repair (Kilkenny et al., 2008). Crb2 was found to be significantly up-regulated in the skeletal muscle at acute and chronic stages. Carbohydrate *N*-acetylgalactosamine-4-O-sulfotransferase 9 (CHST9), a member of the N-acetylgalactosamine-4-O-sulfotransferase family that catalyzes the transfer of sulfate to position 4 of nonreducing terminal GalNAc residues, is implicated in cellular signaling events (Xia et al., 2000; Baenziger, 2003; Zhao et al., 2010). The expression of CHST9 was found to be up-regulated in the acute stage in skeletal muscle. SESN1, which was found to be critical for prolonging the life span of *Caenorhabditis elegans* by preventing muscle degeneration, is required for ROS clearance and plays a key role in defense against HS (Yang et al., 2013). The expression of SESN1 was found to be up-regulated in the skeletal muscle in the acute stage. The NR4A3 is a nuclear orphan receptor and a member of the Nur77 family. The NR4A3 activates several genes that are critical for regulating cell cycle, inflammation, and DNA repair (Wenzl et al., 2015). NR4A3 expression was found to up-regulated in the skeletal muscle of lowland chicken. The differential expression of these key hub genes, which had significant expression correlation with 91 other genes in the network (degrees), suggests that the lowland chickens were affected by HS, and they responded robustly by activating, cell cycle checkpoints, cell cycle arrestors, ROS clearance, and DNA damage and repair mechanisms.

The top hub genes in the highland coexpression network (**Figure 5A**), identified by the degrees of connections, were *COMMD4*, *TTC32*, *H1F0*, and *ACYP1*. COMM (Copper metabolism gene MURR1) domain containing 4 (COMMD4) is an inhibitor of TNF (tumor necrosis factor)–induced NF-κB (nuclear factor κB) (Burstein et al., 2005). Activated NF-κB regulates the expression of several genes that controls cell proliferation, apoptosis, and inflammation (Liu et al., 2016). The expression of COMMD4 was found to be significantly down-regulated in the cardiac (acute and chronic stages) and skeletal muscle (acute stage) of highland chickens, possibly indicating the activation of NF-κB transcription regulatory factor. TTC32 was found to be up-regulated in the cardiac and skeletal muscle tissues at chronic and acute stages, respectively. The function of TTC32 is unknown; however, a number of TPR (tetratricopeptide repeat domain) interact with HSP family HSP70, HSP70, and HSP90 and are required for regulation of protein folding and transport (Ballinger et al., 1999), and HSP90AA1, a member of the HSP90 family, was significantly up-regulated in the skeletal muscle in the acute stage. H1F0 is involved in apoptotic DNA fragmentation (Wang et al., 2018) and contributes to labeling DNA damage (Keck et al., 2018). The expression of H1F0 was found to be up-regulated at both stages in the skeletal muscle and at the chronic stage in cardiac tissue in the highland chickens. The expression of ACYP1, an isoform of ACYP, which can induce apoptosis and is involved in ion transport (Degl’innocenti et al., 2004; Degl’innocenti et al., 2019), was found to be significantly elevated in the cardiac (chronic) and skeletal muscle (acute), while the expression of ACYP2 (another isoform of ACYP) was found to be elevated in the cardiac (Acute) and skeletal muscle (Chronic). This might indicate the activation of apoptosis due to DNA or cellular damage.

Pathway enrichment analysis of the genes in the GCNs (**Figures 5B, D**) showed that the p53 signaling and PPAR signaling pathways were enriched in both the lowland and highland networks. While p53 signaling pathway plays a pivotal role in cell death and cell survival by activating genes that induce cell cycle regulation, DNA repair, and cell death (Vogelstein et al., 2000; Zhang et al., 2010), PPAR signaling is critical for energy homeostasis (Wang, 2010), and considerable energy is spent in maintaining body temperature and dissipating heat, under hyperthermic condition. Moreover, there is a transcriptional dependence on PPAR for heat shock response (Vallanat et al., 2010). The steroid biosynthetic pathway was enriched only in the lowland network (Cook et al., 2015). Genes in this pathway can modulate the activities of RORγ (retinoic acid–related orphan receptors), which can regulate apoptosis (Kurebayashi et al., 2000). The MAPK signaling pathway and protein processing in endoplasmic reticulum (PP-ER) pathway were enriched only in the highland network (**Figure 5B**). HS is proteotoxic, and denatured proteins can become cytotoxic by forming aggregates (Srikanth et al., 2017a). Cell responds to this by activating the PP-ER pathway (Harding et al., 1999), which increases the protein-folding capacity in the endoplasmic reticulum and also activates apoptosis (programmed cell death) (Welihinda et al., 1999). Heat shock is known to activate several members of the MAPK family, which constitutes serine/threonine kinases that play a crucial role in transmitting signals required for cell growth, differentiation, and apoptosis (Pearson et al., 2001; Gorostizaga et al., 2005). The enrichment of multiple apoptosis activation pathways and hub genes that are proapoptotic factors in the highland network suggests that considerable cellular damage has taken place in the highland chickens

## Conclusion

This study examined the transcriptome response to HS of two IC ecotypes from the lowlands and highlands of Kenya. Rectal temperature measurements and RNA-Seq analysis revealed that comparing the responses of acute HS and chronic HS indicated acclimatization of both lowland and highland chickens in such a short period. Furthermore, the response to HS is tissue and stage specific. The GCN analysis showed that the hub genes identified in the lowland chickens were cell cycle arrestors and DNA repair genes, while the highland hub genes were apoptotic and oxidative stress–responsive genes. These results lead us to conclude that, although both the ecotypes experienced HS, the lowland chickens responded more robustly than the highland chickens and might have a higher tolerance to HS than the highland chickens. This better acclimatization may be due to previous adaptation to higher temperatures in the lowland environment. This study extends our understanding of the HS response of chickens, and the genes and pathways identified could serve as a foundation for improving thermal tolerance in chickens.

## Data Availability Statement

The raw reads are available at the NCBI (National Center for Biotechnology Information) SRA (Sequence Read Archive) database under accession number PRJNA557270.

## Ethics Statement

The animal study was reviewed and approved by Institutional Animal Care and Use Committee, National Institute of Animal Science, South Korea.

## Author Contributions

MB, MP, and J-EP conceived the project. SK and DL collected the samples. KS, MB, HK, and WP performed the experiments. MB and J-EP secured the funding for the project. KS, J-MK, and WP analyzed the data. KS and J-EP interpreted the results and drafted the manuscript. J-MK and MP edited the manuscript.

## Funding

This study was carried out with the support of “Investigation of expression profiles and genetic network related to heat stress for chicken” (project PJ01122101) and “Experimental metadata generation for sharing genome and metagenome data of Korean and African chickens” (project PJ01275601), Rural Development Administration (RDA), Republic of Korea. KS and HK were supported by a 2019 RDA Fellowship Program of National Institute of Animal Science, Rural Development Administration, Republic of Korea.

## Conflict of Interest

The authors declare that the research was conducted in the absence of any commercial or financial relationships that could be construed as a potential conflict of interest.
