# Corrigendum: Cardiac and Skeletal Muscle Transcriptome Response to Heat Stress in Kenyan Chicken Ecotypes Adapted to Low and High Altitudes Reveal Differences in Thermal Tolerance and Stress Response

**DOI:** 10.3389/fgene.2020.00197

**Published:** 2020-03-03

**Authors:** Krishnamoorthy Srikanth, Himansu Kumar, Woncheoul Park, Mijeong Byun, Dajeong Lim, Steve Kemp, Marinus F. W. te Pas, Jun-Mo Kim, Jong-Eun Park

**Affiliations:** ^1^Animal Genomics and Bioinformatics Division, National Institute of Animal Science, RDA, Wanju, South Korea; ^2^Animal Biosciences, International Livestock Research Institute (ILRI), Nairobi, Kenya; ^3^Wageningen UR Livestock Research, Animal Breeding and Genomics, Wageningen, Netherlands; ^4^Department of Animal Science and Technology, Chung-Ang University, Anseong, South Korea

**Keywords:** heat stress, hub genes, PPAR signaling, MAPK signaling, p53 signaling, RNA-Seq

In the original article, there was a mistake in **Supplementary Table 1** and **Supplementary Table 2**. The expression values given for *PDK4* in Supplementary Table 1, ALL_M, CLL_M, AHL_M, CHL_M contrasts were −3.90808, 2.10011, −4.12057, and −4.12057 the correct values are −4.1009, 2.07292, −4.63904, and 3.05659 same value should appear at the “Max_expression_level” column in HL_node_table in Supplementary Table 2.

Similarly the expression values of *MT4* given in Supplementary Table 1 for ALL_H, CLL_H, AHL_H, and CHL_H are −1.20147, 1.485881, −1.19557, and 1.0025, the correct values are −3.1675, −1.82983, −1.35669, and −1.84142. To reflect this change, columns “Max_expression_level,” “Max_Tissue,” and “Up/Down” on Supplementary Table 2, “LL_node_table” tab is corrected.

The authors apologize for this error and state that this does not change the scientific conclusions of the article in any way. The original article has been updated.

