# Supplementary figures and images for: Cardiac and Skeletal Muscle Transcriptome Response to Heat Stress in Kenyan Chicken Ecotypes Adapted to Low and High Altitudes Reveal Differences in Thermal Tolerance and Stress Response
Source: Front Genet. 2019 Oct 11;10:993. doi: 10.3389/fgene.2019.00993 (PMC6798392; doi:10.3389/fgene.2019.00993)

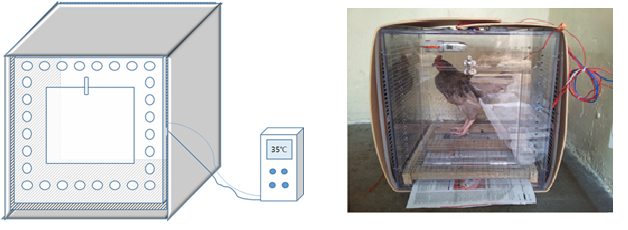

Supplement: Supplementary file 1 [file Image_1.tif]
