# Supplementary material for: Cardiac and Skeletal Muscle Transcriptome Response to Heat Stress in Kenyan Chicken Ecotypes Adapted to Low and High Altitudes Reveal Differences in Thermal Tolerance and Stress Response
Source: Front Genet. 2019 Oct 11;10:993. doi: 10.3389/fgene.2019.00993 (PMC6798392; doi:10.3389/fgene.2019.00993)
Supplement: Supplementary file 4 [file Table_3.docx]

**Summary of mapping and alignment statistics**

| **Region** | **Group** | **Time** | **Samples** | **Tissues** | | | | | | | |
| --- | --- | --- | --- | --- | --- | --- | --- | --- | --- | --- | --- |
|  |  |  |  | **Heart** | | | | **Muscle** | | | |
|  |  |  |  | **Read Pairs** | **Both Surviving** | **Dropped** | **Alignment Rate** | **Read Pairs** | **Both Surviving** | **Dropped** | **Alignment Rate** |
| **Low** | **Control** | **Acute (5 hr)** | **ALL_C_1** | 19729078 | 17877257 (90.61%) | 357477 (1.81%) | 85.43% | 18339429 | 18127114 (98.84%) | 32035 (0.17%) | 84.33% |
|  |  |  | **ALL_C_2** | 21531093 | 20233178 (93.97%) | 189188 (0.88%) | 90.33% | 15292044 | 15080506 (98.62%) | 34497 (0.23%) | 83.46% |
|  |  |  | **ALL_C_3** | 25162577 | 24976415 (99.26%) | 25222 (0.10%) | 91.39% | 19075376 | 18743930 (98.26%) | 38725 (0.20%) | 86.04% |
|  |  |  | **ALL_C_4** | 21111682 | 20100903 (95.21%) | 133382 (0.63%) | 89.17% | 19892496 | 19696025 (99.01%) | 27366 (0.14%) | 87.16% |
|  | **Treatment** |  | **ALL_T_1** | 22542509 | 22300918 (98.93%) | 30722 (0.14%) | 92.77% | 17247672 | 17067976 (98.96%) | 35628 (0.21%) | 83.23% |
|  |  |  | **ALL_T_2** | 19391994 | 19172460 (98.87%) | 26671 (0.14%) | 92.79% | 16490538 | 16340444 (99.09%) | 23801 (0.14%) | 85.04% |
|  |  |  | **ALL_T_3** | 25884717 | 25562828 (98.76%) | 35252 (0.14%) | 91.67% | 22017209 | 21739213 (98.74%) | 42456 (0.19%) | 85.35% |
|  |  |  | **ALL_T_4** | 18795160 | 18601255 (98.97%) | 20513 (0.11%) | 92.10% | 21907451 | 21603298 (98.61%) | 37516 (0.17%) | 86.78% |
| **High** | **Control** |  | **AHL_C_1** | 18694013 | 18425947 (98.57%) | 34592 (0.19%) | 91.62% | 15649777 | 15432863 (98.61%) | 30121 (0.19%) | 85.63% |
|  |  |  | **AHL_C_2** | 19771301 | 18705449 (94.61%) | 140997 (0.71%) | 86.12% | 20913841 | 20596199 (98.48%) | 57712 (0.28%) | 81.50% |
|  |  |  | **AHL_C_3** | 18429646 | 18114165 (98.29%) | 34624 (0.19%) | 92.00% | 19640209 | 19404912 (98.80%) | 25557 (0.13%) | 85.71% |
|  |  |  | **AHL_C_4** | 19433350 | 19217815 (98.89%) | 23732 (0.12%) | 92.37% | 21916130 | 21628254 (98.69%) | 41790 (0.19%) | 86.46% |
|  | **Treatment** |  | **AHL_T_1** | 20292245 | 20069248 (98.90%) | 28742 (0.14%) | 92.15% | 16617801 | 16475988 (99.15%) | 25625 (0.15%) | 85.89% |
|  |  |  | **AHL_T_2** | 20592273 | 20308438 (98.62%) | 41697 (0.20%) | 92.53% | 28228737 | 25376270 (89.90%) | 753321 (2.67%) | 81.63% |
|  |  |  | **AHL_T_3** | 20299612 | 18584098 (91.55%) | 241660 (1.19%) | 85.10% | 19320991 | 17687266 (91.54%) | 343972 (1.78%) | 87.29% |
|  |  |  | **AHL_T_4** | 18385273 | 18165079 (98.80%) | 22268 (0.12%) | 91.77% | 21675525 | 21463005 (99.02%) | 30281 (0.14%) | 84.61% |
| **Low** | **Control** | **Chronic (72 hr)** | **CLL_C_1** | 19791975 | 19561991 (98.84%) | 29650 (0.15%) | 91.91% | 18766868 | 18554656 (98.87%) | 32920 (0.18%) | 86.35% |
|  |  |  | **CLL_C_2** | 20077910 | 19812863 (98.68%) | 33823 (0.17%) | 91.82% | 18321594 | 18094728 (98.76%) | 30867 (0.17%) | 86.19% |
|  |  |  | **CLL_C_3** | 16675959 | 15699603 (94.15%) | 130984 (0.79%) | 89.79% | 23699015 | 23356875 (98.56%) | 51171 (0.22%) | 84.13% |
|  |  |  | **CLL_C_4** | 18754751 | 18564745 (98.99%) | 23350 (0.12%) | 90.87% | 21694524 | 21402543 (98.65%) | 38431 (0.18%) | 86.69% |
|  | **Treatment** |  | **CLL_T_1** | 19816223 | 19594480 (98.88%) | 27215 (0.14%) | 91.82% | 26465995 | 26144645 (98.79%) | 39231 (0.15%) | 85.41% |
|  |  |  | **CLL_T_2** | 21138027 | 20067281 (94.93%) | 151489 (0.72%) | 87.07% | 22897553 | 22653654 (98.93%) | 34759 (0.15%) | 86.11% |
|  |  |  | **CLL_T_3** | 21269731 | 20958239 (98.54%) | 39665 (0.19%) | 91.80% | 20525890 | 20268308 (98.75%) | 34777 (0.17%) | 86.24% |
|  |  |  | **CLL_T_4** | 20847785 | 20508480 (98.37%) | 43971 (0.21%) | 93.21% | 22952715 | 22636480 (98.62%) | 45366 (0.20%) | 85.94% |
| **High** | **Control** |  | **CHL_C_1** | 19352472 | 19155778 (98.98%) | 32577 (0.17%) | 92.63% | 23292339 | 23011175 (98.79%) | 39498 (0.17%) | 85.41% |
|  |  |  | **CHL_C_2** | 20275571 | 20070475 (98.99%) | 29808 (0.15%) | 91.09% | 23853560 | 23486368 (98.46%) | 49646 (0.21%) | 85.54% |
|  |  |  | **CHL_C_3** | 18927021 | 18725297 (98.93%) | 22730 (0.12%) | 91.98% | 20001238 | 18166769 (90.83%) | 376492 (1.88%) | 81.52% |
|  |  |  | **CHL_C_4** | 19200458 | 18969107 (98.80%) | 26193 (0.14%) | 92.19% | 28061044 | 27785126 (99.02%) | 42114 (0.15%) | 85.51% |
|  | **Treatment** |  | **CHL_T_1** | 18679468 | 18439940 (98.72%) | 30083 (0.16%) | 91.22% | 19006596 | 16809261 (88.44%) | 553641 (2.91%) | 86.34% |
|  |  |  | **CHL_T_2** | 18613558 | 18368490 (98.68%) | 30192 (0.16%) | 93.15% | 28456852 | 28154399 (98.94%) | 44472 (0.16%) | 85.09% |
|  |  |  | **CHL_T_3** | 21319044 | 21027263 (98.63%) | 36339 (0.17%) | 92.53% | 22191759 | 21847292 (98.45%) | 54379 (0.25%) | 86.04% |
|  |  |  | **CHL_T_4** | 21842246 | 21549619 (98.66%) | 34840 (0.16%) | 92.74% | 25078963 | 23050307 (91.91%) | 371546 (1.48%) | 81.37% |
